# CT radiomics based on different machine learning models for classifying gross tumor volume and normal liver tissue in hepatocellular carcinoma

**DOI:** 10.1186/s40644-024-00652-4

**Published:** 2024-01-26

**Authors:** Huai-wen Zhang, De-long Huang, Yi-ren Wang, Hao-shu Zhong, Hao-wen Pang

**Affiliations:** 1 Department of Radiotherapy, The Second Affiliated Hospital of Nanchang Medical College, Jiangxi Clinical Research Center for Cancer, Jiangxi Cancer Hospital, 330029 Nanchang, China; 2 Department of Oncology, The third people’s hospital of Jingdezhen, The third people’s hospital of Jingdezhen affiliated to Nanchang Medical College, 333000 Jingdezhen, China; 3 School of Clinical Medicine, Southwest Medical University, 646000 Luzhou, China; 4 School of Nursing, Southwest Medical University, 646000 Luzhou, China; 5 Department of Hematology, Huashan Hospital, Fudan University, 200040 Shanghai, China; 6 Department of Oncology, The Affiliated Hospital of Southwest Medical University, 646000 Luzhou, China

**Keywords:** CT radiomics, Hepatocellular carcinoma, Automatic classification, Machine learning

## Abstract

**Background & aims:**

The present study utilized extracted computed tomography radiomics features to classify the gross tumor volume and normal liver tissue in hepatocellular carcinoma by mainstream machine learning methods, aiming to establish an automatic classification model.

**Methods:**

We recruited 104 pathologically confirmed hepatocellular carcinoma patients for this study. GTV and normal liver tissue samples were manually segmented into regions of interest and randomly divided into five-fold cross-validation groups. Dimensionality reduction using LASSO regression. Radiomics models were constructed via logistic regression, support vector machine (SVM), random forest, Xgboost, and Adaboost algorithms. The diagnostic efficacy, discrimination, and calibration of algorithms were verified using area under the receiver operating characteristic curve (AUC) analyses and calibration plot comparison.

**Results:**

Seven screened radiomics features excelled at distinguishing the gross tumor area. The Xgboost machine learning algorithm had the best discrimination and comprehensive diagnostic performance with an AUC of 0.9975 [95% confidence interval (CI): 0.9973–0.9978] and mean MCC of 0.9369. SVM had the second best discrimination and diagnostic performance with an AUC of 0.9846 (95% CI: 0.9835– 0.9857), mean Matthews correlation coefficient (MCC)of 0.9105, and a better calibration. All other algorithms showed an excellent ability to distinguish between gross tumor area and normal liver tissue (mean AUC 0.9825, 0.9861,0.9727,0.9644 for Adaboost, random forest, logistic regression, naivem Bayes algorithm respectively).

**Conclusion:**

CT radiomics based on machine learning algorithms can accurately classify GTV and normal liver tissue, while the Xgboost and SVM algorithms served as the best complementary algorithms.

## Introduction

Liver cancer is one of the most common cancers worldwide [1]. The liver is also one of the most common metastatic organs for other carcinomas, including metastases related to colorectal, melanoma, and pancreatic cancers [2–6]. Currently, palliative treatment for hepatocellular carcinoma includes radiofrequency ablation, transarterial chemoembolization, immunotherapy, and gene therapy in addition to traditional radiotherapy (RT) and chemotherapy [7]. RT is an important part of clinical oncology treatment. It also plays a significant role in the treatment of hepatocellular carcinoma. RT utilizes various radiation devices and radionuclides to kill tumor cells. Determining the tumor and normal tissue outline during delineating is an important step in the process of RT, directly affecting its accuracy [8]. The differences in clinical experience and different doctors’ opinions may cause sizeable differences in the delineation of target areas between different radiologists. Furthermore, the delineation process is time-consuming and labor-intensive. Manual sketching of target areas and crucial organs for a patient with a tumor can take the clinician hours to accomplish to ensure that the maximum dose is administered according to the delineated tumor areas and that the normal tissue is protected as much as possible. On the other hand, the patient’s weight, as well as morphology and size of the target area and organs, may change during the treatment process. Therefore, the automatic outline of important organs is particularly valuable. The automatic organ delineation methods can be implemented using the active contours model. This model uses a priori knowledge of the target shape to define a closed curve around the region to be outlined in the form of a parametric equation with an energy function. The equation converges to the boundary contours of the target by taking advantage of the high gradient position of the image and smoothness and continuity of the curve [9–11]. Atlas-based library and alignment methods can also outline certain medical images based on the relative spatial location and shape of the normal organs inside the body that are similar even for different populations. Therefore, beginners can follow the expert atlas to obtain the same features in the coarse image texture.Pre-sketched computed tomography (CT) images (atlas + markers) can be used to assist in creating new sketches [12]. Deep learning automatic delineation techniques with self-learning capability can extract complex hierarchical features from images. The basic idea of deep learning methods is to represent abstract information through multiple high-level features. The methods for medical image outlining include convolutional neural network [13], fully convolutional network [14], and U-Net [15] methods. In the image texture-based method, radiomics features extracted from the image using texture parameters can provide information about the spatial arrangement of image pixels. The intensity arrangement of the image texture can be quantified and image features can be extracted to divide the image region into meaningful parts such as normal organs and tissues [16]. Lambin et al. first introduced the concept of radiomics in 2012 as a field focused on improving image analysis and high-throughput extraction of a large number of quantitative features [17]. Radiomics features of tumor regions have been reported to have an important predictive role in the classification of hepatocellular carcinoma [18–19]. However, there are still relatively few studies using texture features to outline the gross tumor areas in hepatocellular carcinoma. It is clinically important to improve the effectiveness and accuracy of distinguishing between normal liver and target areas for RT by analyzing the radiomics features. Relevant studies have reported using radiomics for classification, while identification prognosis studies have extracted MRI or CT image histological features based on patients diagnosed with liver cancer [20–23]. In a radiomics study on non-enhanced CT in hepatocellular carcinoma (HCC) by Zhao et al., the machine-learning algorithm, such as SVM, was effective in classifying benign and malignant liver lesions in the test set, obtaining an AUC of 0.8990 and an overall accuracy of 0.8400 [24].

Therefore, it is possible that a machine-learning classification algorithm that distinguishes gross tumor tissue from normal tissue based on the image features extracted from the CT images before RT can produce a significant guidance and reference value for confirmation of liver GTV in RT for hepatocellular carcinoma patients. In addition, there is an error rate associated with hepatocellular carcinoma delineation by low-grade radiotherapists. The machine-learning algorithm that distinguishes normal tissue from tumor tissue is likely to improve their accuracy in outlining target areas [25–26].There are fewer automatic outlining software packages for liver tumors in relevant automatic target area outlining software. Therefore, the extracted relevant CT image radiomics feature values were used in the present study for the differentiation of normal tissues from RT target in order to achieve automatic outlining of RT target and improve the treatment effect.

## **Materials and methods**

### **Patient population**

The clinical data and CT images for 104 patients with hepatocellular carcinoma who underwent liver biopsy surgery between January *2016* and September *2022* were included in the study. Inclusion criteria were as follows: (1) patients who underwent a preoperative two-phase enhanced scan; (2) no history of RT and chemotherapy; (3) malignant liver tumors were diagnosed by postoperative pathology and had complete clinical data; and (4) patients aged 18–80 years with no contraindications for RT and life expectancy greater than three months. Exclusion criteria were as follows: (1) images with severe motion artifacts or evident noise; (2) maximum tumor diameter of < 1.0 cm; (3) other tumor diseases; and (4) pregnant or breastfeeding females and individuals who refused to use suitable contraception. The baseline clinical data distribution is described in Table 1.

**Table 1:** Baseline of 104 enrolled patients from clinical center

SEX	(%)	Female	19 (18.4)
	(%)	Male	85 (81.6)
Age	(years,mean (SD))		55.59 (10.58)
ECOG			1.17 (3.09)
PT	(second,mean (SD))		13.91 (2.12)
AFP	(IU/ml,mean (SD))		4,161.75 (6,977.57)
TBIL	(µmol/L,mean (SD))		29.03 (36.27)
ALB	(g/L,mean (SD))		36.51 (5.38)
ALT	(U/L,mean (SD))		66.13 (97.89)
AST	(U/L,mean (SD))		115.58 (313.87)
WBC	(10^9/L,mean (SD))		5.59 (2.43)
HB	(g/L,mean (SD))		123.38 (24.85)
PLT	(10^9/L,mean (SD))		143.41 (80.18)
Liver cirrhosis	(%)	NO	34 (32.0)
	(%)	YES	70 (68.0)
Hepatitis B virus	(%)	NO	30 (28.2)
	(%)	YES	74 (71.8)

### **Image acquisition**

Images for histology analysis were obtained from RT localization CT scans. Enhanced CT scans were performed using a LightSpeed RT 16 CT machine (GE Healthcare, Chicago, IL, USA). The scan parameters were set as follows: tube voltage, 120 kVp; field of view, 250–400 mm; pixel size, 512 × 512; and layer thickness, 2.5 mm. CT images were preprocessed by wavelet-based filtering methods before extraction of histological features. The GTV and normal liver tissue were regarded as the regions of interest representing the target area for RT and normal tissue, respectively.

### **Feature extraction and downscaling**

For feature extraction, all CT images and regions of interest were batched and converted to nii format. Manual segmentations were performed with oversight from an experienced radiologist with > 10 years of experience in radiology therapy. Feature extraction was based on Python 3.9 implemented using the pyradiomics software (http://PyRadiomics.readthedocs.io/en/latest). All images were resampled according to a voxel size of 1 × 1 × 1 mm^3^ before feature extraction. The image quantification method used bin widths of 25. The GTV and normal liver tissue were regarded as the regions of interest. The radiomics features included the First Order Statistics (firstorder), Gray Level Cooccurence Matrix (glcm), Gray Level Dependence Matrix (gldm), Gray Level Run Length Matrix (glrlm), Gray Level Size Zone Matrix (glszm), and Neighbouring Gray Tone Difference Matrix (ngtdm). Shape features were abandoned. These algorithms for obtaining radiomics features were referenced from the Image Biomarker Standardization Initiative [27]. The radiomics feature dimensionality reduction and selection using the least absolute shrinkage and selection operator (LASSO) regression model with five-fold cross-validation were used to select features with nonzero coefficients, such that we could choose the variables with the smallest mean square error. All feature selection procedures were executed on the training cohort and used for the test cohort. The variance inflation factor (VIF)measures the severity of multicollinearity in multiple regression models. It represents the ratio of the estimator variance of the regression coefficient to the variance when no linear correlation between the independent variables is assumed. The VIF can be calculated as follows:

$$ \text{V}\text{I}\text{F}=\frac{1}{1-{\text{R}}_{\text{i}}^{2}}$$

where *Ri* is the negative correlation coefficient of the regression analysis for other independent variables. The larger the VIF, the greater the possibility of collinearity between the independent variables. Generally, multicollinearity is assumed when the VIF value is greater than five. Thus, removing the radiomics features with a VIF value greater than five is necessary. Z-score transformation was utilized for field correction and intensity standardization for each feature.

### **Construction of matching learning algorithm-based classification models and evaluation of diagnostic efficacy**

All statistical assessments were carried out using R-4.1.1 software. The final selected features were utilized to construct the radiomics models. To select a classifier model with the greatest recognition of GTV, six mainstream machine learning algorithm training models were chosen, which included logistic regression, SVM, random forest, Xgboost, Adaboost, and naive Bayes algorithms, respectively. The diagnostic performances of the six models were compared using the AUC of the receiver operating characteristic curve (ROC), sensitivity, specificity, positive prediction value (PPV), negative prediction value (NPV), and Matthews correlation coefficient (MCC). The best radiomics model was then screened.

$$\text{M}\text{C}\text{C}=\frac{\text{T}\text{P}\cdot \text{T}\text{N}-\text{F}\text{P}\cdot \text{F}\text{N}}{sqrt( (\text{T}\text{P}+\text{F}\text{P})\cdot (\text{T}\text{P}+\text{F}\text{N})\cdot (\text{T}\text{N}+\text{F}\text{P})\cdot (\text{T}\text{N}+\text{F}\text{N}))}$$

Five-fold cross-validation and grid search were used to tune and optimize the model hyperparameters for Adaboost (mfinal = 10, maxdepth = 3), Xgboost (“eta"=0.05, “max_depth"=6, “colsample_bytree"=1, “min_child_weight"=1, “subsampl e"=0.73, “gamma"=1, “lambda"=1, “alpha"=0, “max_delta_step"=0, “colsample_bylevel"=1) and random forest (Mtry = 2, nodesize = 15, sampsize = 131, importance = TRUE). Other parameters remained at a default level.

### **Discrimination and calibration evaluation of multiple machine learning algorithms models**

Calibration plot and AUC reflect how accurately a model can predict the type of true labels. Calibration curves depict the calibration of each model in terms of the agreement between the predicted probabilities of observed outcomes (GTV area). The y-axis for these curves represents the actual rate of the GTV area, the x-axis shows the predicted GTV area probabilities, and the diagonal dotted line describes a perfect prediction by an ideal model.

## Results

### **Radiomics feature screening and collinearity analysis**

**Fig. 1:**
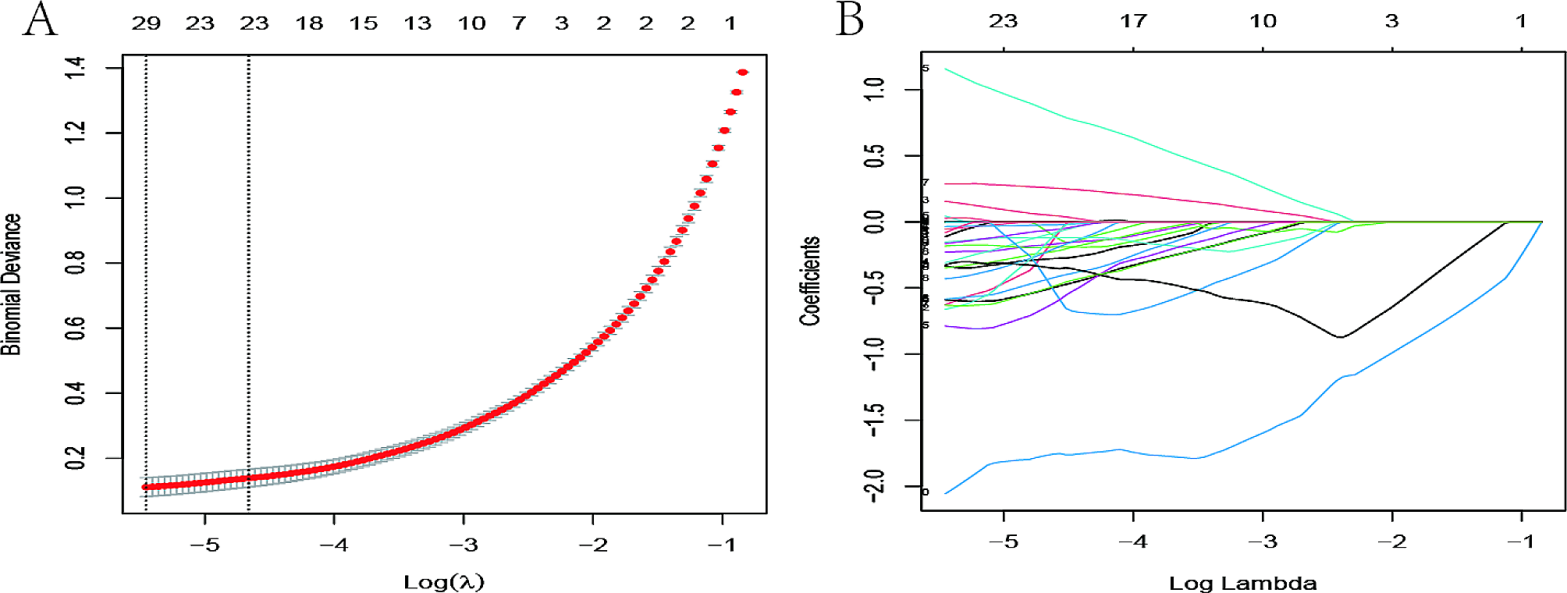
Radiomics feature selection with the least absolute shrinkage and selection operator (LASSO) binary logistic regression model. (A) Tuning parameter (l) selection in the LASSO model used five-fold cross-validation with minimum criteria. Left vertical lines indicate the optimal value of the LASSO tuning parameter (λ). (B) LASSO coefficient profile plot with different log (λ). Vertical dashed lines represent 29 radiomics features with nonzero coefficients selected with the optimal λ value

A total of 104 patients (208 samples) diagnosed with HCC were enrolled in the study, and 1,395 radiomics features were extracted from GTV and normal liver tissue. Among them, 29 features with nonzero coefficients were retained after LASSO logistic regression analysis for variable screening. According to this calculation, the model’s deviation was the smallest when the minimum value was 0.004292374. Figure 1 shows the binomial deviance and lambda (λ). The VIF of the independent variable in the logistic regression model was calculated after screening the variables. The variables with VIF > 5 after the LASSO logistic regression analysis were removed by eliminating all highly co-linear features that could lead to model overfitting as previously described until the logistic model converged. A total of seven radiomics features were retained as a result. The VIFs were all < 5, which indicates that no multicollinearity existed among the seven radiomics features (Table 2).

**Table 2:** Model collinearity analysis

Variables	VIF
lbp.2D_glrlm_RunLengthNonUniformity	1.169
square_firstorder_Kurtosis	1.432
squareroot_firstorder_RootMeanSquared	1.606
wavelet.LLH_firstorder_Median	1.305
wavelet.LLH_firstorder_TotalEnergy	2.241
wavelet.HHH_glszm_LowGrayLevelZoneEmphasis	1.292
wavelet.LLL_glszm_SizeZoneNonUniformity	2.229

### **Comparing diagnosis efficiency of different machine learning algorithms**

**Fig. 2:**
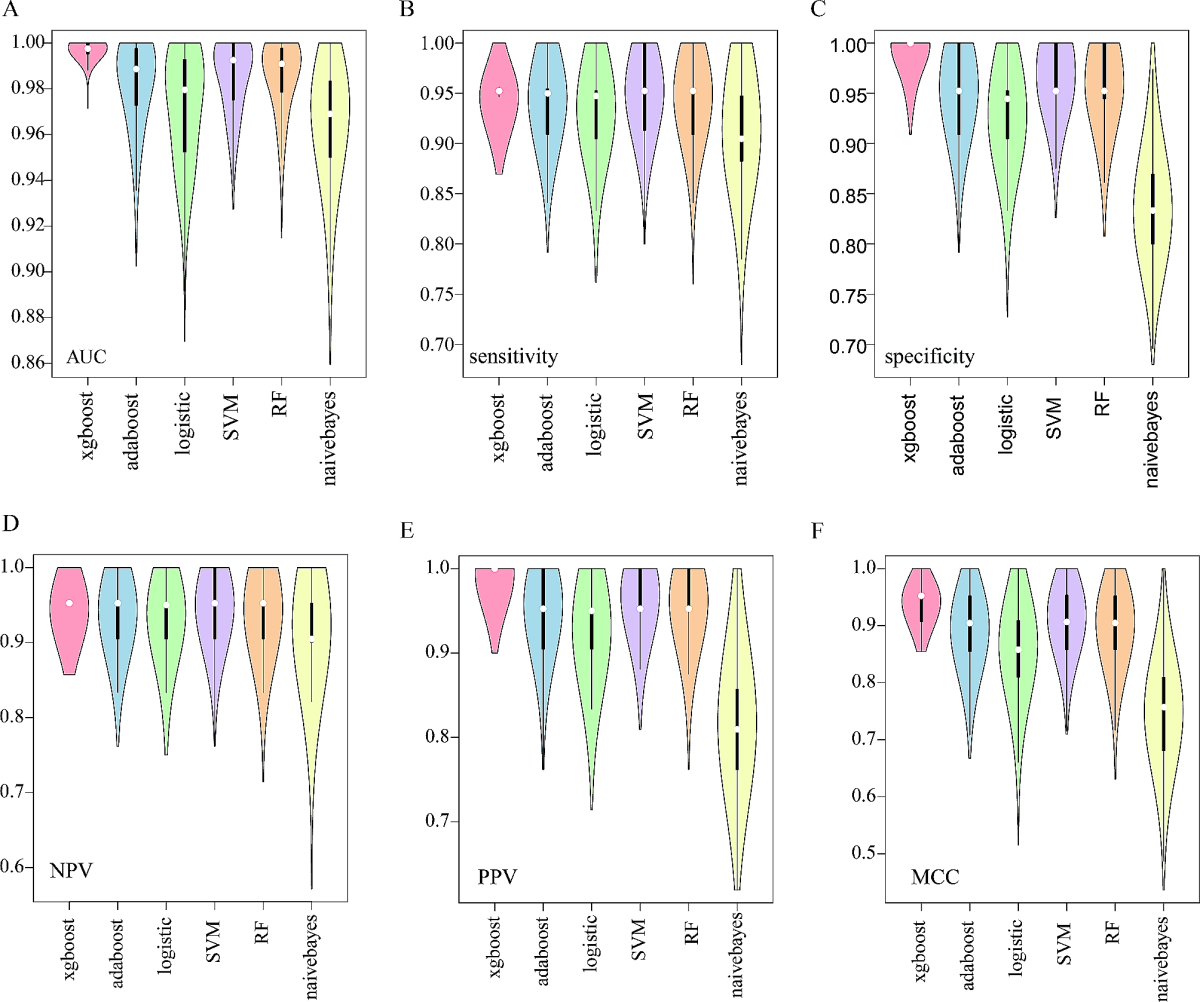
(A-F)Violin plots for different machine learning algorithms with 200 iterations of five-fold cross-validation for AUC, accuracy, specificity, NPV, PPV, and MCC

The data were divided 4:1 (167:41), yielding training and validation groups using five-fold cross-validation. The process was iterated 200 times with different initialization seeds, generating a total of 1,000 modeling data points for each machine learning algorithm. The training and validation groups were separately normalized before model construction and validation. Among the evaluation indicators, Xgboost achieved the best performance than other algorithms with a mean AUC of 0.9978. Xgboost also had the highest mean specificity of 0.9921, mean PPV of 0.9918, and mean MCC of 0.9369, SVM with mean specificity 0.9490 performed better than other algorithms and had an mean NPV of 0.9468 in the validation groups. Naive Bayes was the worst algorithm to distinguish between the GTV and normal liver. The violin diagrams show that the Xgboost values were more concentrated at a better level. However, the other algorithms were more widely distributed. The better the algorithm, the higher the concentration, showing its robustness (Fig. 2).

### Discrimination and calibration evaluation of different machine learning algorithms

**Fig. 3:**
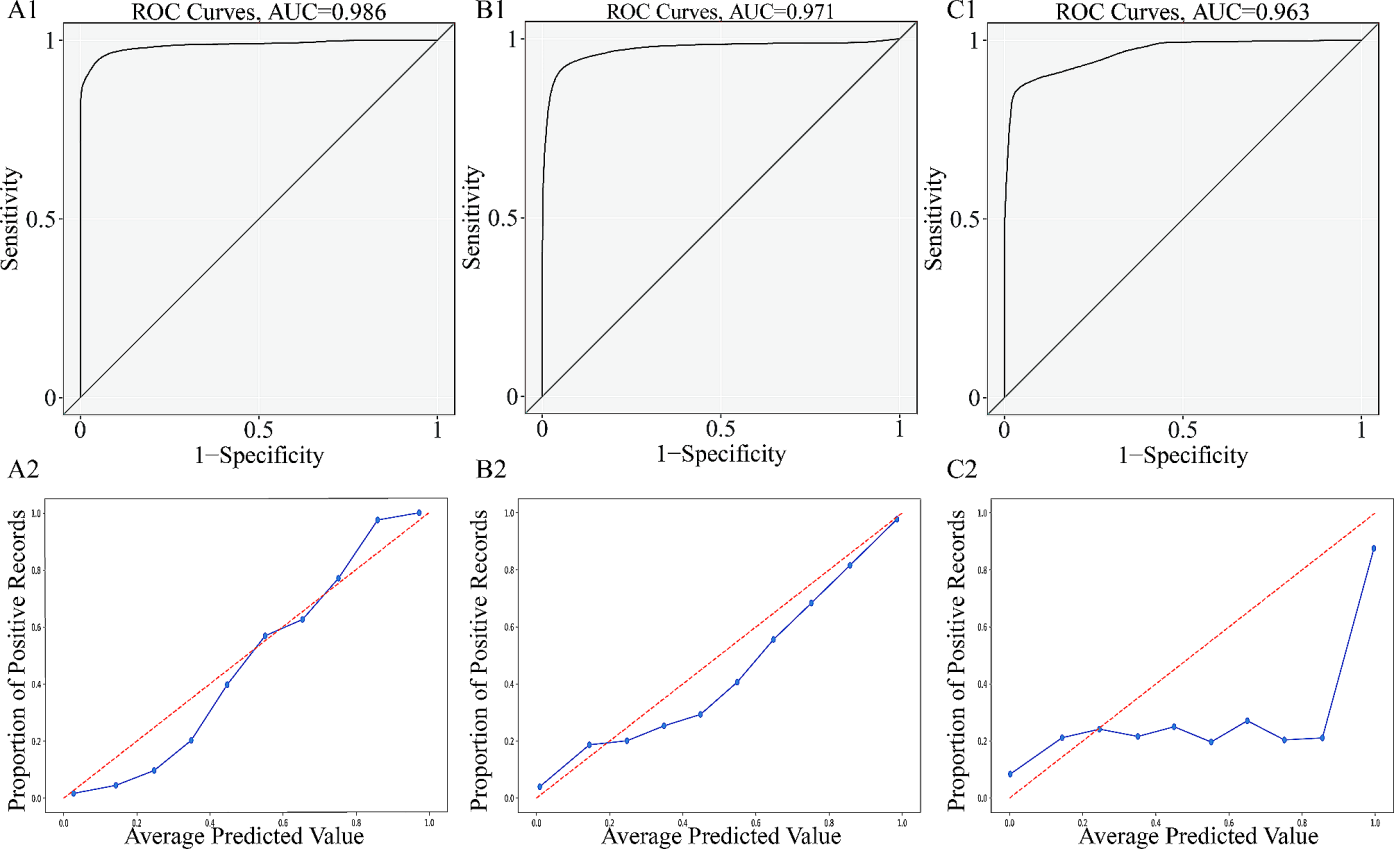
(A1–C1) Receiver operating characteristic curves for Xgboost, Adaboost and SVM models in the validation group. (A2–C2) Calibration plots for Xgboost, Adaboost and SVMmodels in the validation group

**Fig. 4:**
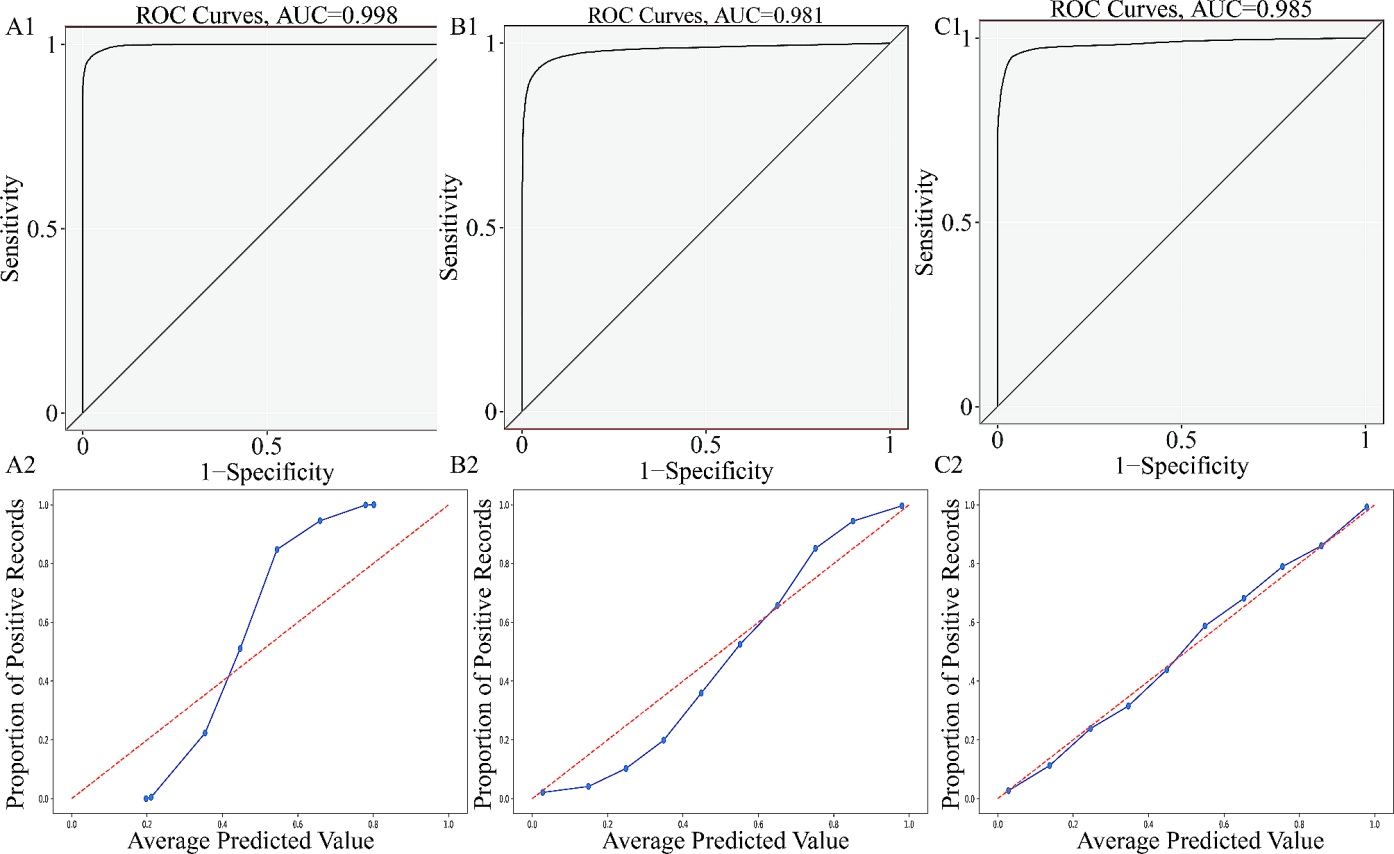
(A1–C1) Receiver operating characteristic curves for random forest, logistic regression, and naive Bayes models in the validation group. (A2–C2) Calibration plot for random forest, logistic regression, and naive Bayes models in the validation group

Five-fold cross-validation with 200 iterations and different initialization seeds produced 41,000 validations and predictions. The predicted probability and true classification were used for each prediction in the validation group as a fusion estimator for AUC calculation and generation of the calibration plot. In a fused evaluation of the ROC curves, Xgboost had the highest discrimination with an AUC of 0.9975 (95% confidence interval (CI): 0.9973–0.9978), followed by random forest with an AUC of 0.9856 (95% CI: 0.9846–0.9866). Similar performance was achieved by SVM with an AUC of 0.9846 (95% CI: 0.9835–0.9857), followed by Adaboost with an AUC of 0.9812 (95% CI: 0.9799–0.9825). Naive Bayes remained the worst model with an AUC of 0.9633 (95% CI: 0.9617–0.9649). SVM fit well in terms of calibration curves, while Xgboost did not perform well. The predicted value for SVM was approximately equal to the observed value, and the blue line overlapped with the reference line. For Xgboost and Adaboost, when the positive observation rate was < 50%, the predicted value was lower than the observed value, the risk was underestimated, and the blue line was above the reference line. When the positive observation rate was > 50%, the predicted value was greater than the observed value, the risk was overestimated, and the blue line was above the reference line. Logistic and naive Bayes models were prone to underestimating the positive observation rate. Brier scores for Xgboost, Adaboost, SVM, RF, logistic regression, and naive Bayes were 0.0620, 0.0480, 0.0370, 0.0440, 0.057, and 0. 1170, respectively. The diagnostic efficiency of different machine learning algorithms in the training and validation groups is shown in Tables 3 and 4. The ROC curve and calibration plot for the machine learning models in the validation groups are shown in Figs. 3 and 4.

**Table 3:** Diagnosis efficiency of different machine learning algorithms in training groups

	AUC	Sensitivity	Specificity
Adaboost	0.9973(0.9962,0.9975,0.9986)	0.9748(0.9647,0.9762,0.9878)	0.9804(0.9750,0.9875,0.9880)
Xgboost	0.9945(0.9911,0.9954,0.9969)	0.9612(0.9529,0.9643,0.9647)	0.9747(0.9747,0.9756,0.9759)
SVM	0.9935(0.9915,0.9928,0.9955)	0.9632(0.9529,0.9639,0.9643)	0.9625(0.9524,0.9639,0.9643)
RF	0.9975(0.9972,0.9987,0.9994)	0.9855(0.9762,0.9880,1.0000)	0.9860(0.9765,0.9880,1.0000)
logistic	0.9880(0.9847,0.9867,0.9904)	0.9522(0.9398,0.9512,0.9634)	0.9457(0.9398,0.9412,0.9524)
naivebayes	0.9732(0.9692,0.9720,0.9765)	0.9222(0.9143,0.9178,0.9296)	0.8174(0.7959,0.8061,0.8280)
	NPV	PPV	MCC
Adaboost	0.9744(0.9639,0.9759,0.9880)	0.9803(0.9759,0.9880,0.9881)	0.9549(0.9402,0.9524,0.9759)
Xgboost	0.9605(0.9518,0.9639,0.9643)	0.9750(0.9759,0.9759,0.9762)	0.9357(0.9280,0.9398,0.9407)
SVM	0.9632(0.9524,0.9639,0.9643)	0.9624(0.9518,0.9639,0.9643)	0.9257(0.9157,0.9277,0.9398)
RF	0.9853(0.9759,0.9880,1.0000)	0.9858(0.9759,0.9880,1.0000)	0.9713(0.9639,0.9759,0.9880)
logistic	0.9524(0.9398,0.9518,0.9639)	0.9453(0.9398,0.9405,0.9518)	0.8978(0.8797,0.8923,0.9159)
naivebayes	0.9336(0.9277,0.9286,0.9398)	0.7900(0.7590,0.7738,0.8072)	0.7316(0.7003,0.7184,0.7445)

#Data outside and inside the brackets indicate the mean and first, median, and third quartiles of the results, respectively, for five-fold cross-validation iterated 200 times.

**Table 4:** Diagnosis efficiency of different machine learning algorithms in validation groups

	AUC	Sensitivity	Specificity
Adaboost	0.9825(0.9728,0.9887,0.9976)	0.9408(0.9091,0.9500,0.9545)	0.9487(0.9091,0.9524,1.0000)
Xgboost	0.9978(0.9952,0.9976,1.0000)	0.9453(0.9500,0.9524,0.9524)	0.9921(1.0000,1.0000,1.0000)
SVM	0.9856(0.9751,0.9925,1.0000)	0.9490(0.9130,0.9524,1.0000)	0.9629(0.9500,0.9524,1.0000)
RF	0.9861(0.9786,0.9909,0.9977)	0.9439(0.9091,0.9524,0.9545)	0.9541(0.9444,0.9524,1.0000)
logistic	0.9727(0.9524,0.9796,0.9929)	0.9319(0.9048,0.9474,0.9524)	0.9275(0.9048,0.9444,0.9524)
naivebayes	0.9644(0.9500,0.9690,0.9833)	0.9129(0.8824,0.9048,0.9474)	0.8391(0.8000,0.8333,0.8696)
	NPV	PPV	MCC
Adaboost	0.9385(0.9048,0.9524,0.9524)	0.9473(0.9048,0.9524,1.0000)	0.8876(0.8548,0.9045,0.9523)
Xgboost	0.9447(0.9524,0.9524,0.9524)	0.9918(1.0000,1.0000,1.0000)	0.9369(0.9069,0.9524,0.9524)
SVM	0.9468(0.9048,0.9524,1.0000)	0.9623(0.9524,0.9524,1.0000)	0.9105(0.8581,0.9065,0.9535)
RF	0.9417(0.9048,0.9524,0.9524)	0.9532(0.9500,0.9524,1.0000)	0.8964(0.8581,0.9048,0.9524)
logistic	0.9303(0.9048,0.9500,0.9524)	0.9250(0.9048,0.9500,0.9524)	0.8574(0.8095,0.8581,0.9089)
naivebayes	0.9176(0.9000,0.9048,0.9524)	0.8197(0.7619,0.8095,0.8571)	0.7445(0.6807,0.7571,0.8095)

#Data outside and inside the brackets indicate the mean and first, median, and third quartiles of the results, respectively, for five-fold cross-validation iterated 200 times.

## Discussion

In recent years, artificial intelligence technologies, such as machine learning and neural networks, have been widely used in the field of automatic tumor and organ delineation in RT. These include automatic segmentation technology based on CNN and automatic classification technology based on the U-NET model. These methods have greatly reduced clinician workload and raised working efficiency [27–31]. According to Patel et al., however, deep learning methods in automatic organ delineation may fail due to many factors, such as domain shift [32], adversarial noise, low image quality, and robustness problems if the input images have a different distribution from the training datasets for the regress network. In a radiomics study, a high number of features (typically more than a hundred) are extracted characterizing a given ROI in different ways [33]. The features are then tested as prognosticators. Moreover, features have to be carefully selected based on their robustness and sensitivity towards the delineation process to be clinically applicable. This, in turn, reveals another potential use for radiomics features and distributions as a possible application for radiomics-based generation of regions/volumes of interest (ROI/VOI) with certain characteristics to improve anatomic auto-contouring. To date, only a small number of studies have been performed to assess the usability of radiomics features in the quantification of contouring precision [34]. There have been no studies systematically evaluating various machine learning algorithms applied in the classification of RT targets for hepatocellular carcinoma to date.

By building different models with image texture, the present study suggested that CT-based radiomics with machine leaning may effectually differentiate between normal tissue and GTV making it feasible and reliable in clinical practice of radiomics radiologists. It can also provide evidence for automatic delineation of hepatocellular carcinoma with radiomics features for RT target areas. The generalization ability of the classifiers was also evaluated in order to prevent overfitting and achieved similar results as the validator in the training and validation groups. The present study found that the Xgboost algorithm maintained a good discrimination in terms of accuracy, PPV, MCC, and AUC in the fused ROC curve analysis in the validation groups. However, Xgboost performed poorly in the calibration curve analysis. The calibration curve had a sigmoid shape, which was caused by the model’s lack of confidence. Its predicted probability was always relatively close to 0.5. The second-best algorithm was SVM, which achieved the highest score in sensitivity and NPV and ranked second for MCC. MCC takes into account true and false positives and false negatives and is usually considered to be a balanced measure that produces high scores only if the prediction obtains good results in all four confusion matrix categories (TP, TN, FN, and FP). In summary, since both linear and nonlinear models achieved good results in the validation groups, the radiomics features of GTV and normal liver tissue were considered as linearly separable data. Artificial intelligence models have achieved great success in the automatic delineation of organs [35–36], but the accuracy of the automatic delineation of tumor regions remains a problem. In the present study, the robustness of the algorithms was verified via a large number of iterations and each model was compared in detail in terms of its diagnostic performance, discrimination, and calibration of six leading and popular machine learning algorithms for differentiating GTV areas in hepatocellular carcinoma. Therefore, if we can use radiomics features extracted through the training set instead of pixel values to distinguish between tumor and normal liver regions, this may be a supplement to the depth learning automatic delineation technology. The region of interest can be limited to the entire liver region, and extract only the radiomics features extracted through the training set to improve efficiency.

## Conclusion

CT radiomics based on machine learning algorithms can accurately classify GTV and normal liver tissue, while the Xgboost and SVM algorithms served as the best complementary algorithms. Therefore, multiple machine-learning methods were first used in the present study for the differentiation of gross tumor volume and normal liver tissue in order to achieve automatic outlining of RT target areas in the future and improve the treatment effect of RT and the efficiency of radiotherapists.

### Limitations

This study has some limitations. This study mainly focus on the study of GTV of hepatocellular carcinoma (HCC)and normal liver, not for all types of liver tumors. In the future, we will prospectively collect patients with different types of liver cancer, including liver metastases, for multi-center research, and further evaluate the bleeding in the tumor or the surrounding tissues by combining with the deep learning algorithm.

## Data Availability

All data generated and analyzed during this study are included in this published article.
